# Calcified Amorphous Tumor of Left Ventricle: A Rare Cardiac Tumor

**DOI:** 10.7759/cureus.17908

**Published:** 2021-09-12

**Authors:** Rupesh Kumar, Vikram Halder, Soumitra Ghosh, Basant Kumar, Subhrashis Guha Neogi, Balamurugan Thirunavukkarasu, Amanjit Bal

**Affiliations:** 1 Cardiothoracic Surgery, Postgraduate Institute of Medical Education and Research, Chandigarh, IND; 2 Cardiology, Postgraduate Institute of Medical Education and Research, Chandigarh, IND; 3 Anaesthesiology, Postgraduate Institute of Medical Education and Research, Chandigarh, IND; 4 Pathology, Postgraduate Institute of Medical Education and Research, Chandigarh, IND

**Keywords:** non-neoplastic mass, calcified amorphous tumor, echocardiography, eosinophilic amorphous material, rare tumour

## Abstract

Cardiac calcified amorphous tumor (CAT) is a rare, non-neoplastic, intra-cavity cardiac mass. Only a few cases have been described in the literature.

A 46-year-old Indian female presented with decompensated heart failure. On echocardiography, 1.9 x 1.7 cm pedunculated mobile mass in the left ventricle attached to the intraventricular septum was seen. On cardiac magnetic resonance imaging (MRI), the lesion was isointense. Histopathology of the excised mass revealed fibrin deposition with eosinophilic amorphous material in the center with the periphery of the lesion showing calcification without any myxomatous tissue. A final diagnosis of CAT of the heart was established.

CAT is composed of calcium deposits in the background of amorphous degenerating fibrinous material. It presents as a pedunculated mass in any chamber of the heart with a very high preponderance of distal embolization. Differentiation from calcified atrial myxoma, calcified thrombi, or other cardiac neoplasms is very difficult. Histopathological examination is the mainstay of diagnosis. Treatment is emergency excision to prevent distal embolization.

CAT is a rare non-neoplastic tumor, which is mainly a tissue diagnosis after its resection.

## Introduction

The cardiac calcified amorphous tumor (CAT) is a rare non-neoplastic cardiac mass that is composed of amorphous fibrinous material and calcium. This tumor was first described by Reynolds and colleagues [1]. The most common site is the mitral annulus followed by the right atrium (RA), right ventricle, left ventricle (LV), left atrium, and the tricuspid annulus [2]. Clinicopathologically it mimics calcified myxoma and calcified thrombi, and on transthoracic echocardiography, it is very difficult to distinguish between these entities. Patients may present with dyspnea (due to obstruction) or clinical features of distal embolization. Echocardiography is the primary modality for diagnosis but histopathological examination is the gold standard. It appears as a pedunculated calcified mass on echocardiography and computed tomography (CT). Size may vary from small punctate lesion to very large mass. Cardiac magnetic resonance imaging (MRI) shows low signal intensity on T1 and T2 without post gadolinium contrast enhancement. An embolic event is a frequent presenting condition and mobile lesions definitely indicate higher embolic risks. So early surgery is needed followed by histopathological confirmation.

## Case presentation

A 46-year-old female patient presented to the emergency with decompensated heart failure and decreased urine output. She is a known case of chronic kidney disease (stage 4) on medication and one episode of thromboembolic cerebrovascular accident (right frontoparietal infarct) two months ago.

**Figure 1:**
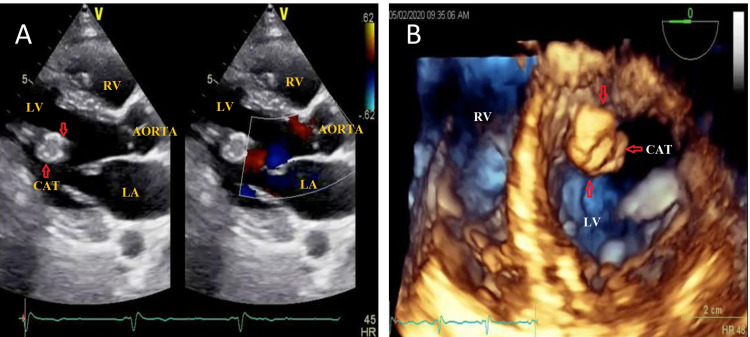
A: Two-dimensional echocardiography, PLAX view showing 1.9 x 1.7 cm pedunculated mass in the LV. B: Three-dimensional echocardiography showing 1.9 x 1.7 cm pedunculated mobile mass in LV attached to interventricular septum. LV, left ventricle; RV, right ventricle; LA, left atrium; CAT, calcified amorphous tumor.

**Figure 2:**
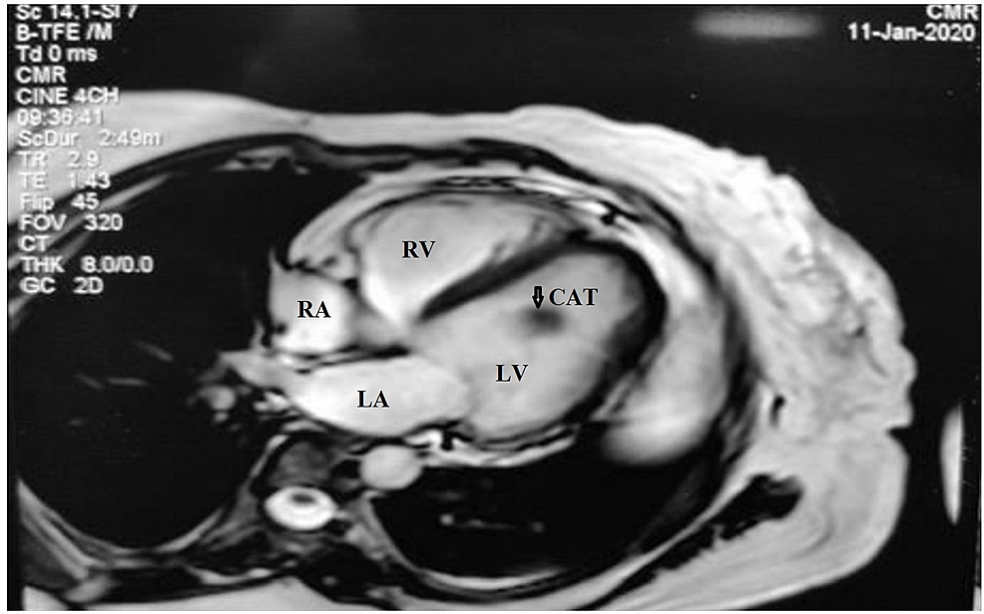
Cardiac T2-weighted magnetic resonance imaging showing isointense mass in the LV attached to endocardium of the interventricular septum through a narrow pedicle. LV, left ventricle; LA, left atrium; RA, right atrium; RV, right ventricle; CAT, calcified amorphous tumor.

She was stabilized with medical treatment. On echocardiography (Figure 1), she had global hypokinesia with an ejection fraction (EF) of 35-40%. She was diagnosed with heart failure with reduced EF (HFrEF). Echocardiography also revealed a 1.9 x 1.7 cm pedunculated mobile mass in the LV, which was attached to the intraventricular septum. On cardiac MRI (Figure 2) isointense lesion in the LF was attached to the endocardium through a narrow pedicle. As per institutional protocol coronary angiography was done, and on angiography, it was noted that the mid part of the left anterior descending (LAD) artery was 70-80% stenosed and the posterior descending artery (PDA) was 80% stenosed.

**Figure 3:**
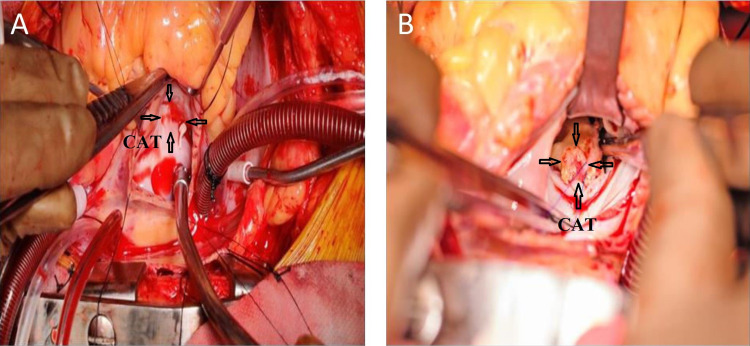
A: Tail of the mass in the left ventricle attached to the interventricular septum (trans right atrial approach). B: Mass in left ventricle after retracting the tricuspid leaflet. CAT, calcified amorphous tumor.

The patient was taken up for surgery (Figure 3). After sternotomy, left internal mammary artery (LIMA) and right-sided great saphenous vein (RSVG) were harvested as the caliber of RSVG was adequate and more than left-sided great saphenous vein assessed preoperatively. After heparinization, aorto bicaval cannulation was done and a cross-clamp was applied. The RA was opened and the inter-atrial septum (IAS) was incised. The LV was approached through RA and IAS as the left atrium and interatrial groove were adhered to the pericardium. Then LV mass was resected and IAS and RA were closed. RSVG was anastomosed to PDA and the ascending aorta and LIMA was anastomosed to LAD. Then gradually the cross-clamp was taken off and decannulation was done. After introducing intercostal drains and pacing wire, the sternum was closed. Immediately after the operation, ventilation time was 12 hours and the vasoactive inotropic score was 28. Intensive care unit stay and hospital stays were four days and seven days, respectively.

**Figure 4:**
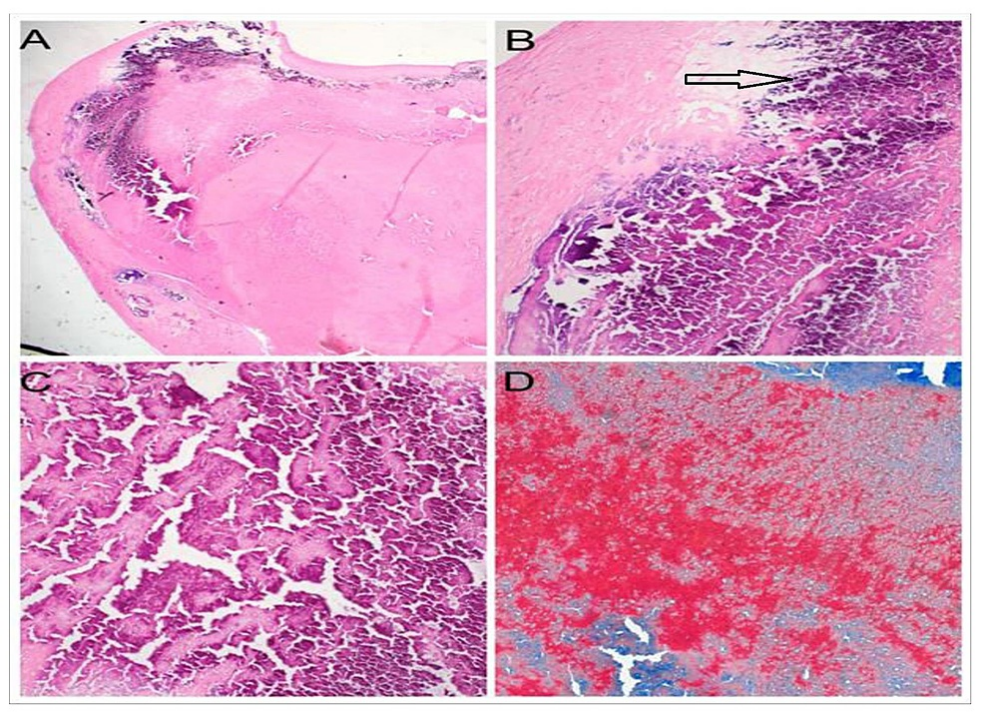
A: Low-magnification view of calcified amorphous tumor. There is fibrin deposition with eosinophilic amorphous material in the center (H&E 10X). B: Periphery of the lesion showing bluish calcification (H&E 40X). C: Focal ossification highlighted by grungy amphophilic material (H&E 40X). D: Martius Scarlet Blue stain highlighting the fibrin as red color (40x).

Histopathology of the tumor revealed fibrin deposition with eosinophilic amorphous material in the center with the periphery of the lesion showing calcification. No myxomatous tissue was seen (Figure 4).

On six-month follow-up, there was no recurrent mass but had persistent LV systolic dysfunction with function class of New York Heart Association II and on optimal medical treatment.

## Discussion

The primary cardiac tumor is rare and the most common tumor is atrial myxoma. CAT is a rare cardiac mass. This tumor is more common in females than in males. Most of the affected patients are of the fifth decade.

Pathogenesis of the tumor is the formation of a thrombus due to hypercoagulability and abnormal calcium and phosphorus metabolism. The growth rate of CATs is slow but the growth rate of CATs associated with mitral annular calcification is fast. The most common location is the mitral annulus, which is commonly due to previous mitral annular calcification and is commonly seen in end-stage renal disease. Other affected chambers of the heart according to incidence are the RA, right ventricle, LV, left atrium, and tricuspid annulus [2].

The most common presenting symptom is dyspnea on exertion due to obstruction of the blood flow. Other presenting features are angina, syncope (due to distal embolism), pulmonary embolism, systemic embolism, and, rarely, a patient may be diagnosed incidentally. Other associated diseases are valvular heart disease, end-stage renal disease, and hyperparathyroidism. Chances of embolic events are more in CATs with mitral annular calcification [3].

On echocardiography, CAT is described as pedunculated calcified mass. Size may vary from small punctate lesion to very large mass. Myxoma is a mobile mass and 20 % of atrial myxoma are calcified. Cardiac fibroma is calcified intramyocardial mass in which commonly LV is involved. Other causes of cardiac calcification are end-stage renal disease and thrombus [4]. On CT scan or MRI, CATs are either irregular, ovoid, triangular, spherical, or tubular. On configuration, mass is either polypoid or infiltrative with or without a broad base and distribution of calcification is partial or diffuse. On cardiac MRI, homogeneous appearance with low signal intensity on T1- and T2-weighted spin-echo sequences without post gadolinium contrast enhancement in early and delayed sequences was noted [5].

Patients are treated with operative management. After midline sternotomy aorto bicaval or aorto atrial cannulation is done according to the chamber involved. Right atrial and right ventricular lesions are approached through the RA. Left atrial lesions are approached through the left atrium or RA. Left ventricular lesions are approached through the left atrium. Ventricular approaches are not preferred because of the increased chance of ventricular tachycardia. On histopathological examination deposition of heterogeneous calcium with surrounding amorphous eosinophilic and fibrinous material is found [6]. The immediate postoperative outcome is good with very little chance of recurrence [2].

## Conclusions

The CAT is a rare tumor. Early detection and management are needed to prevent complications. There is less chance of recurrence. Since clinical and echocardiographical presentation is similar, histopathological diagnosis is mandatory.
